# A Case of a Huge Iliopsoas Abscess that Perforated and Communicated with the Ureter

**DOI:** 10.31662/jmaj.2023-0217

**Published:** 2024-06-03

**Authors:** Yusuke Tabata, Masato Yanagi, Kazuo Enomoto, Satoshi Omori, Noriyoshi Murotani, Osamu Mitsuhashi, Mitsunori Yasuda, Yuki Sawano, Koichiro Omori, Yoichiro Tabata, Tokifumi Majima

**Affiliations:** 1 Department of Orthopedic Surgery, Mitsuhashi Meisei Hospital, Chiba, Japan; 2 Department of Urology, Nippon Medical School Hospital, Tokyo, Japan; 3 Hemodialysis Department, Mitsuhashi Meisei Hospital, Chiba, Japan; 4 Department of Orthopedic Surgery, Nippon Medical School Hospital, Tokyo, Japan

**Keywords:** iliopsoas abscess, ureter, perforation

## Abstract

We experienced a rare case in which iliopsoas abscess (IPA), caused by an Extended Spectrum β-Lactamase (ESBL)-producing Proteus mirabilis, perforated and communicated with the ureter and caused sepsis. An 84-year-old woman, bedridden due to sequelae of a cerebral hemorrhage, was brought to our hospital with a chief complaint of fever lasting for 3 weeks. Computed tomography (CT) revealed a huge 180 × 110 × 100 mm IPA in the right iliopsoas muscle. The ureter was also found to communicate with the iliopsoas muscle abscess, ureteral stenosis was detected at the same site, and dilatation of the renal pelvis occurred above the area of the ureteral stenosis, indicating hydronephrosis. Considering the mechanism of this case, if the ureter first ruptures and urine leaks, followed by the formation of an IPA, urine will flow along the surrounding fatty tissue and cause an abscess around the ureter and kidney. However, because almost no abscess was detected around the ureter, the abscess was thought to have originated from the iliopsoas muscle located near the center of the ureter. In summary, in this case, an abscess first formed within the iliopsoas muscle, which gradually expanded and compressed the right ureter, resulting in hydronephrosis. The upper ureter, which had become dilated and thinned due to ureteral obstruction, became even more fragile because of the spread of inflammation from the IPA, and the IPA perforated and communicated with the ureter. In patients who have difficulty communicating, the diagnosis of IPA may be delayed because the only symptom is fever. As in this case, if the diagnosis is delayed, the abscess may become large and perforate the ureter; thus, IPA should always be considered as a cause of fever of unknown origin.

## Introduction

Iliopsoas abscess (IPA) is a disease that forms an abscess within the iliopsoas muscle. It is categorized into primary abscess, where the primary focus is unknown and the infection is hematogenous or lymphatic, and secondary abscess, where the infection directly spreads from adjacent organs. Antibiotic treatment as well as surgical incision and drainage are the basic treatment strategies ^(1), (2)^, but recently, percutaneous drainage under computed tomography (CT) or echo guidance has been widely used to reduce invasion. It has been shown to exert a therapeutic effect equivalent to incision and drainage ^(3)^.

We experienced a case in which IPA, caused by an ESBL-producing *Proteus mirabilis*, perforated the ureter and caused sepsis. The patient underwent percutaneous drainage and received antibiotic treatment. We report this case along with a review of the literature.

## Case Report

**Figure 1.**
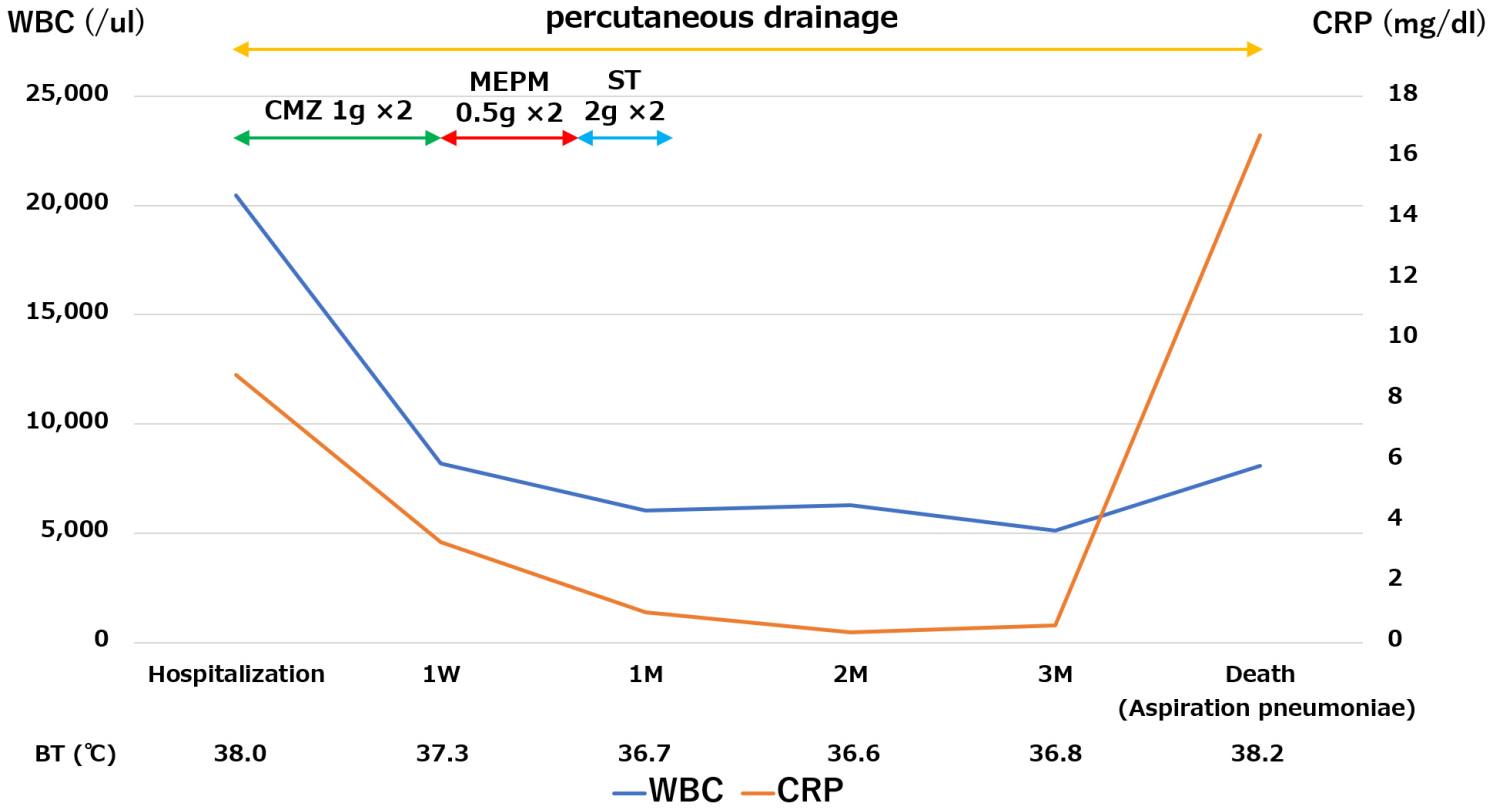
Clinical course of the patient.

**Figure 2.**
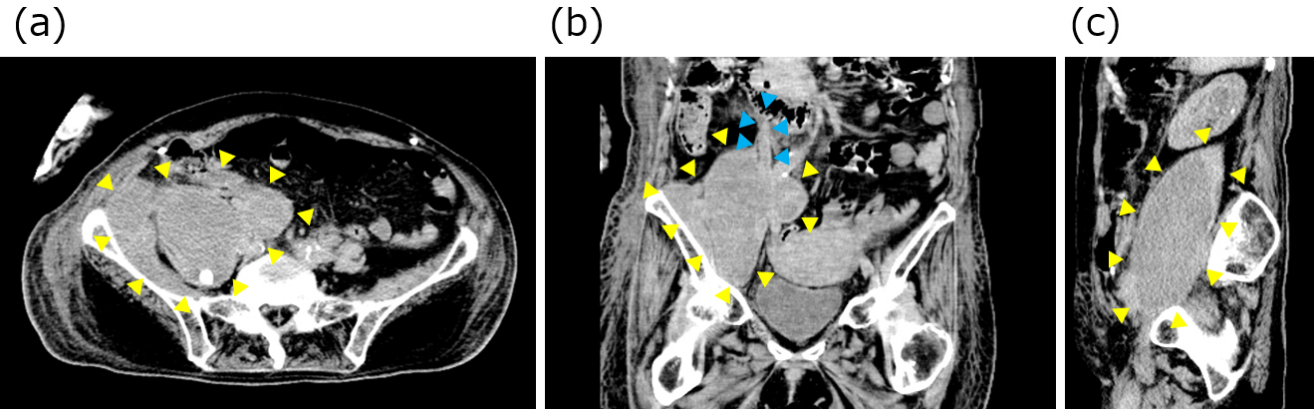
Abdominal CT at admission. The yellow triangular arrow indicates the IPA and the blue triangular arrow indicates the ureter. Communication between the ureter and the iliopsoas abscess was observed. (a) Axial view. (b) Coronal view. (c) Sagittal view.

An 84-year-old woman, bedridden due to sequelae of a cerebral hemorrhage, was brought to our hospital with a chief complaint of fever lasting for 3 weeks. Upon admission, her temperature was 38℃. Blood test revealed high inflammatory response with white blood cell count of 15,510/μl and C-reactive protein of 9.98 mg/dL (Figure 1). In addition, CT revealed a huge 180 × 110 × 100 mm IPA in the right iliopsoas muscle. The ureter was also found to communicate with the iliopsoas muscle abscess, ureteral stenosis was detected at the same site, and dilatation of the renal pelvis occurred above the area of the ureteral stenosis, indicating hydronephrosis. One stone measuring 9 × 6 × 6 mm was detected in the kidney, and one stone measuring 18 mm × 10 cm × 9 mm was found in the IPA (Figure 2).

**Figure 3.**
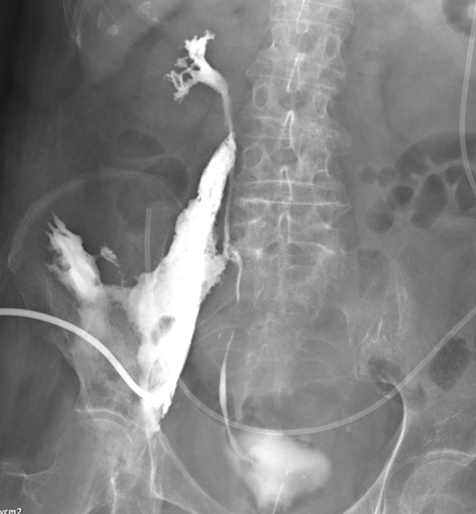
X-ray fluoroscopy. When a contrast medium was injected through the IPA drain, the kidneys, ureter, and bladder were also contrasted.

On the day of admission, percutaneous drainage was performed under echocardiography, and the drainage tube was left in place to drain the abscess fluid. The abscess area was contrasted from the renal pelvis to the ureter and bladder, and traffic between the IPA area and the ureter was confirmed (Figure 3). The collected pus was submitted for culture, and administration of cefmetazole (CMZ) 1 g twice a day via intravenous injection was initiated. *Proteus mirabilis* (extended-spectrum β-lactamase; ESBL) was detected in the urine and abscess cultures. Considering the susceptibility of the bacteria, the antibiotic was changed from CMZ to meropenem hydrate (MEPM) 0.5 g twice a day from the 7th day of hospitalization.

After administering MEPM for 2 weeks, the patient was switched to oral sulfamethoxazole and trimethoprim for another 2 weeks, but systemic drug eruption due to antibiotics was observed; thus, antibiotic administration was discontinued after a total of 4 weeks.

Because of the patient’s advanced age and being bedridden, it was determined that radical surgery (such as ureteric stent insertion and open surgical repair of ureteral perforation) would be difficult; thus, drainage was continued. She remained relatively stable, but she developed aspiration pneumonia on the 119th day of hospitalization and died on the 126th day.

## Discussion

We experienced a very rare case in which IPA perforated and communicated with the ureter. Although many complications of ureteral perforation in surgery for ureteral stones (e.g., transurethral lithotripsy) have been reported, ureteral stones themselves have little chance of causing ureteral perforation. To the best of our knowledge, this is the first report of a case in which the IPA perforated and communicated with the ureter and the ureteral stone migrated to the IPA.

Considering the mechanism of this case, if the ureter first ruptures and urine leaks, followed by the formation of an IPA, urine will flow along the surrounding fatty tissue and cause an abscess around the kidney. However, because almost no abscess was detected around the ureter, the abscess was thought to have originated from the iliopsoas muscle located near the center of the ureter.

**Figure 4.**
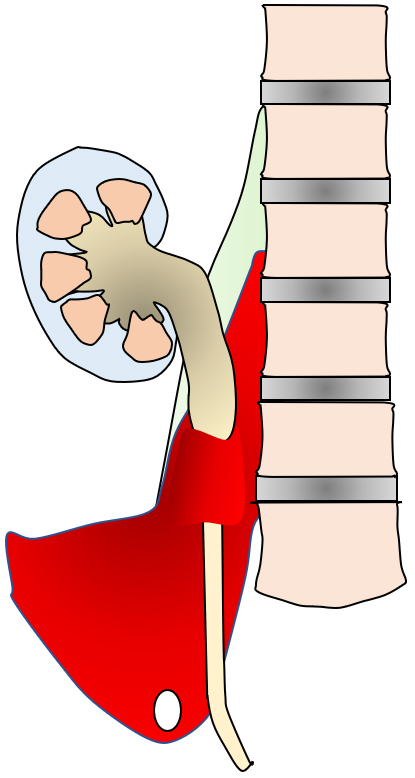
Schema of the patient. The red area indicates IPA. There is a stone in IPA, and IPA causes hydronephrosis.

In summary, in this case, an abscess first formed within the iliopsoas muscle, which gradually expanded and compressed the right ureter, resulting in hydronephrosis. The upper ureter, which had become dilated and thinned due to ureteral obstruction, became even more fragile because of the spread of inflammation from the iliopsoas muscle abscess, and the IPA finally perforated and communicated with the ureter. Subsequently, it was assumed that the stone in the ureter had moved into the IPA (Figure 4). The basis for this is that the stone in the IPA had a round shape, characteristic of common ureteral stones, and that the CT value was similar to that of the right kidney stone.

## Article Information

### Conflicts of Interest

None

### Author Contributions

Yusuke Tabata and Kazuo Enomoto treated the patient. Yusuke Tabata wrote the manuscript. Satoshi Omori, Noriyoshi Murotani, Osamu Mitsuhashi, Mitsunori Yasuda, Yuki Sawano, Koichiro Omori, and Yoichiro Tabata provided useful advice for the manuscript. Masato Yanagi and Tokifumi Majima provided major contributions to the writing of the manuscript. All authors read and approved the final manuscript.

### Approval by Institutional Review Board (IRB)

Not applicable.

### Informed Consent

Informed consent was obtained from the patient.
